# A particular silent codon exchange in a recombinant gene greatly influences host cell metabolic activity

**DOI:** 10.1186/s12934-015-0348-8

**Published:** 2015-10-05

**Authors:** Natalie Rahmen, Christian D. Schlupp, Hitoshi Mitsunaga, Alexander Fulton, Tita Aryani, Lara Esch, Ulrich Schaffrath, Eiichiro Fukuzaki, Karl-Erich Jaeger, Jochen Büchs

**Affiliations:** AVT, Biochemical Engineering, RWTH Aachen University, Worringerweg 1, 52074 Aachen, Germany; Department of Plant Physiology, RWTH Aachen University, Worringerweg 1, 52056 Aachen, Germany; Department of Biotechnology, Graduate School of Engineering, Osaka University, 2-1 Yamada-oka, Suita, 565-0871 Japan; Institute for Molecular Enzyme Technology, Heinrich-Heine-University Düsseldorf, Forschungszentrum Jülich, 52426 Jülich, Germany; Institute of Bio- and Geosciences IBG-1: Biotechnology, Forschungszentrum Jülich GmbH, 52426 Jülich, Germany

**Keywords:** Recombinant protein production, *Escherichia coli*, Silent codon exchange, Synonymous codon, Respiration activity, Metabolic burden, Plasmid copy number

## Abstract

**Background:**

Recombinant protein production using *Escherichia coli* as expression host is highly efficient, however, it also induces strong host cell metabolic burden. Energy and biomass precursors are withdrawn from the host’s metabolism as they are required for plasmid replication, heterologous gene expression and protein production. Rare codons in a heterologous gene may be a further drawback. This study aims to investigate the influence of particular silent codon exchanges within a heterologous gene on host cell metabolic activity. Silent mutations were introduced into the coding sequence of a model protein to introduce all synonymous arginine or leucine codons at two randomly defined positions, as well as substitutions leading to identical amino acid exchanges with different synonymous codons. The respective *E. coli* clones were compared during cultivation in a mineral autoinduction medium using specialized online and offline measuring techniques to quantitatively analyze effects on respiration, biomass and protein production, as well as on carbon source consumption, plasmid copy number, intracellular nucleobases and mRNA content of each clone.

**Results:**

Host stain metabolic burden correlates with recombinant protein production. Upon heterologous gene expression, tremendous differences in respiration, biomass and protein production were observed. According to their different respiration activity the *E. coli* clones could be classified into two groups, Type A and Type B. Type A clones tended to higher product formation, Type B clones showed stronger biomass formation. Whereas codon usage and intracellular nucleobases had no influence on the Type-A–Type-B-behavior, plasmid copy number, mRNA content and carbon source consumption strongly differed between the two groups.

**Conclusions:**

Particular silent codon exchanges in a heterologous gene sequence led to differences in initial growth of Type A and Type B clones. Thus, the biomass concentration at the time point of induction varied. In consequence, not only plasmid copy number and expression levels differed between the two groups, but also the kinetics of lactose and glycerol consumption. Even though the underlying molecular mechanisms are not yet identified we observed the astonishing phenomenon that particular silent codon exchanges within a heterologous gene tremendously affect host cell metabolism and recombinant protein production. This could have great impact on codon optimization of heterologous genes, screening procedures for improved variants, and biotechnological protein production processes.

**Electronic supplementary material:**

The online version of this article (doi:10.1186/s12934-015-0348-8) contains supplementary material, which is available to authorized users.

## Background

Despite an increasing availability of expression systems which can be genetically manipulated [1], *Escherichia coli* is still the preferred prokaryotic host organism for recombinant protein production [2, 3]. Mainly due to a high gene dosage effect, plasmid-based expression is commonly applied [1]. A prevalent system for heterologous gene expression in *E.coli* is based on the T7 RNA polymerase under control of the *lac* UV5 promoter [4]. For inducing recombinant protein production, autoinduction is often preferred over conventional IPTG induction. Advantages are the lower costs and the higher biocompatibility of the inducing compound lactose [2]. Furthermore, autoinduction does not require manual inducer addition since it is controlled by the host’s metabolism. Thus, cell growth and subsequent protein formation are automatically separated from each other [5]. Autoinduction media were developed by Studier [6] and contain a carbon source mixture of glucose, glycerol, and lactose. During consumption of the preferred carbon source glucose, protein formation is suppressed [7]. After glucose depletion, lactose and glycerol are taken up more or less simultaneously. Whereas lactose is partially converted into allolactose [8–10], the physiological inducer of the *lac* operon [5], glycerol is consumed as energy source. Due to their defined chemical composition [11] mineral autoinduction media allow a detailed understanding of metabolic processes during induction and protein production.

A major issue during recombinant protein production is the metabolic burden: the exhaustion of energy and biomass precursors as amino acids or nucleotides from host cell metabolism for synthesizing recombinant material [12–15]. Besides the production of plasmid-encoded proteins, also the plasmids themselves impose a metabolic load on the host cell since cellular resources are required for plasmid replication [1, 16–18]. Furthermore, the different frequency of synonymous codons in foreign coding DNA and host, namely codon bias, can massively affect heterologous protein production levels [19] and *E. coli* growth rates [20]. Prevalent responses of host-cell physiology due to metabolic burden are a decrease in growth rate [21, 22] and change in respiration. Strong variations in respiration activity of the respective *E. coli* clones were observed during recombinant protein production of related human model proteins from fetal brain tissue [22] and the production of identical target proteins only differing by single amino acid exchanges [23]. Thus, respiration activity not only depends on recombinant protein production, but also on small differences between the expressed heterologous genes.

The aim of this study was to investigate the influence of silent codon exchanges within a heterologous gene on production level and metabolic activity of the host *E. coli* BL21(DE3). Therefore, *E. coli* BL21(DE3) clones expressing the wild type gene *lipA* of *Bacillus subtilis* encoding the lipase BSLA [24] and its variants differing only in a single silent codon exchange were examined. Specifically, the wild type codons for arginine and leucine were replaced at two randomly defined positions by all possible synonymous codons. Arginine (Arg107, amino acid position 107) and leucine (Leu143, amino acid position 143) were investigated since both amino acids are encoded by six different codons yielding in a higher number of different sequences for our investigations without altering the wild type amino acid sequence. In addition, clones containing the same amino acid exchange encoded by different synonymous codons were analyzed. To enable online product monitoring, the target protein BSLA was fused to a flavin-based fluorescent protein (FbFP) derived from the light, oxygen, voltage (LOV) domain of the *Bacillus subtilis* YtvA photoreceptor (LOV tag) [25, 26]. As measuring systems, two small-scale online monitoring devices, namely respiration activity monitoring system (RAMOS) and a microtiter plate based cultivation system (BioLector) were applied. RAMOS [27, 28] determines the oxygen transfer rate (OTR) as a characteristic parameter for metabolic activity of the bacteria, whereas the BioLector [29, 30] measures scattered light and fluorescence to trace biomass and protein formation. Furthermore, transcript abundance and intracellular nucleobases were analyzed to gain deeper insights into metabolic processes.

## Results and discussion

Within this study, the influence of silent codon exchanges in a heterologous gene on host cell metabolic activity during recombinant production of an identical protein was investigated. At two positions of the amino acid sequence of the recombinant BSLA, wild type amino acids arginine (Arg107, amino acid position 107) and leucine (Leu143, position 143) were each encoded by all six possible synonymous codons. The investigated *E. coli* clones were named after the respective codon within the DNA sequence. In addition, *E. coli* clones with the amino acid exchanges S56P, D91R, G93Y, S167P, K170E, and G175F caused by the usage of at least two different synonymous codons were analyzed.

### Cultivation under non-inducing conditions

**Fig. 1:**
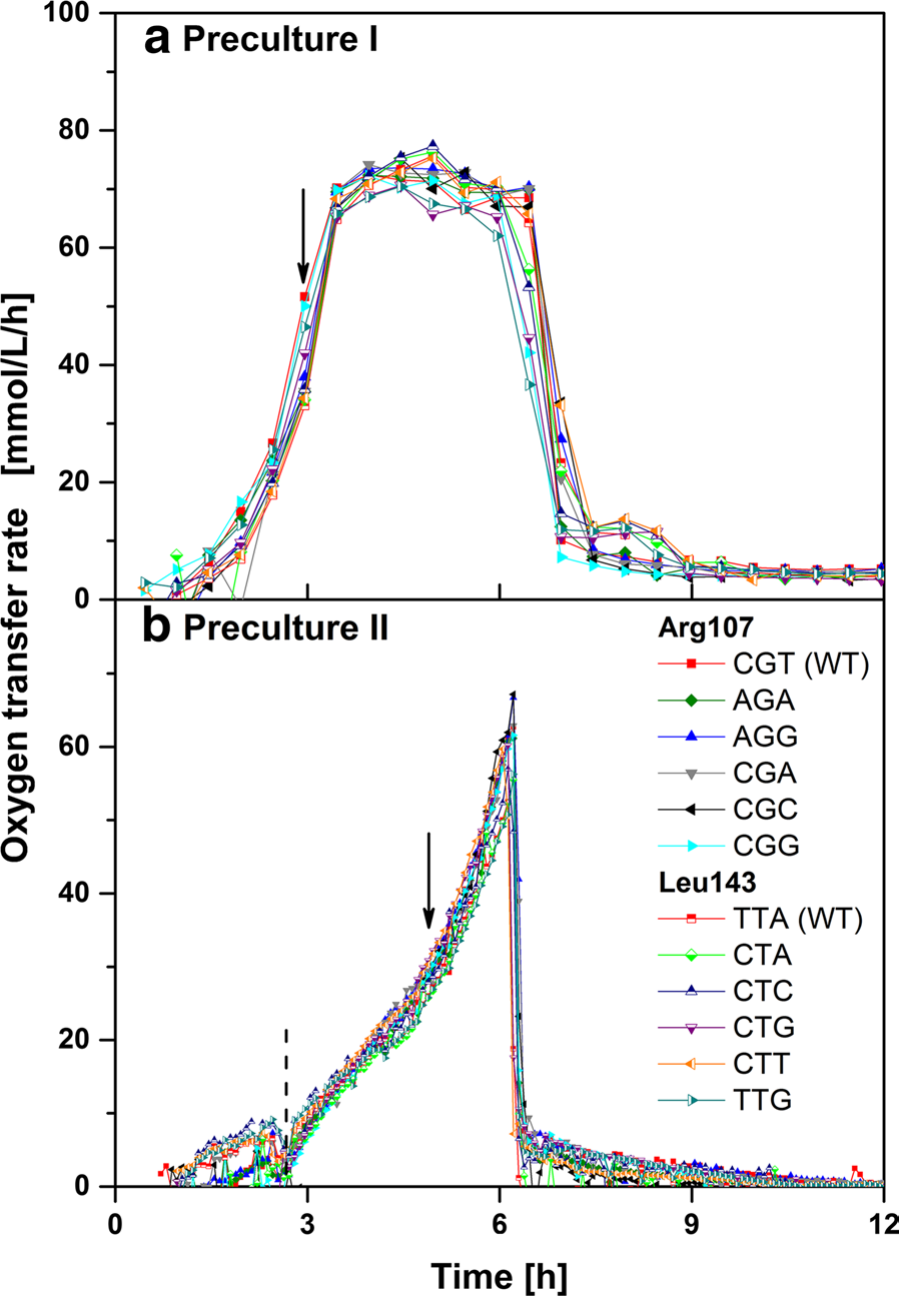
Respiration activity of 12 *E. coli* BL21(DE3) clones grown under non-inducing conditions. Oxygen transfer rates (determined using a RAMOS device) of six clones varying in Arg107 codons and six clones varying in Leu143 codons as a function of time for:** a** first preculture performed in complex TB medium, and b second preculture performed in modified Wilms-MOPS mineral medium containing 0.5 g/L glucose and 5 g/L glycerol. The *arrows* indicate the time points the cultures were harvested and used for inoculating the next cultivation step. The *dotted line* in** b** highlights the depletion of glucose. For the second preculture, a computational analysis of the raw data of the RAMOS device according to Hansen et al. [63] was performed providing an increased density of data points over time. Cultivation conditions: 37 °C, 250 mL flasks, filling volume 10 mL, shaking frequency 350 rpm, and shaking diameter 50 mm

To enable a comparison of the respiration behavior of the investigated *E. coli* clones under reference conditions, precultivations were carried out under non-inducing conditions to prevent recombinant protein production. Precultivations were sequentially performed, first, in complex TB medium to allow strong biomass formation and, second, in modified Wilms-MOPS mineral medium to promote adaptation to main cultivation conditions. Figure 1 presents the OTR of all clones during first and second precultivation.

According to the typical respiration behavior of *E. coli* in TB medium [22, 23, 31, 32], the OTR of all clones increases exponentially until reaching a maximum OTR (Fig. 1a). The horizontal plateau indicates oxygen limitation, the final drop in OTR the depletion of all original carbon sources. Slight differences in the following OTR progress can be attributed to slight differences in the medium composition and the consumption of complex medium components [33]. The selected time point to harvest the cultures for inoculation of the second precultures is during exponential growth after 3 h (black arrow in Fig. 1a). During the second precultivation in Wilms-MOPS mineral medium, the OTR of all clones initially increases due to growth on the preferred carbon source glucose. Slight differences during this phase are due to some signal noise at the rather low oxygen transfer rates at the beginning of the fermentation. Glucose depletion is indicated by a drop in OTR after 2.5 h (dotted line in Fig. 1b). Further exponential increase in OTR up to a maximum is caused by growth on glycerol. The subsequent sharp drop in OTR is due to depletion of all carbon sources. The selected time point to harvest the cultures for inoculation of the main cultures is during exponential growth after 5 h (black arrow in Fig. 1b).

Under non-inducing conditions, all investigated *E. coli* clones depict a quite similar respiration behavior in both, complex and mineral cultivation media. These results perfectly agree with those from cultivations of *E. coli* BL21(DE3) without plasmid and non-induced *E.coli* clones encoding *B. subtilis* lipase A (BSLA) differing in single amino acids under identical cultivation conditions [23]. As a result, the silent codon exchanges of the investigated *E. coli* clones do not have any impact on respiration under non-inducing conditions.

### Cultivation under inducing conditions

**Fig. 2:**
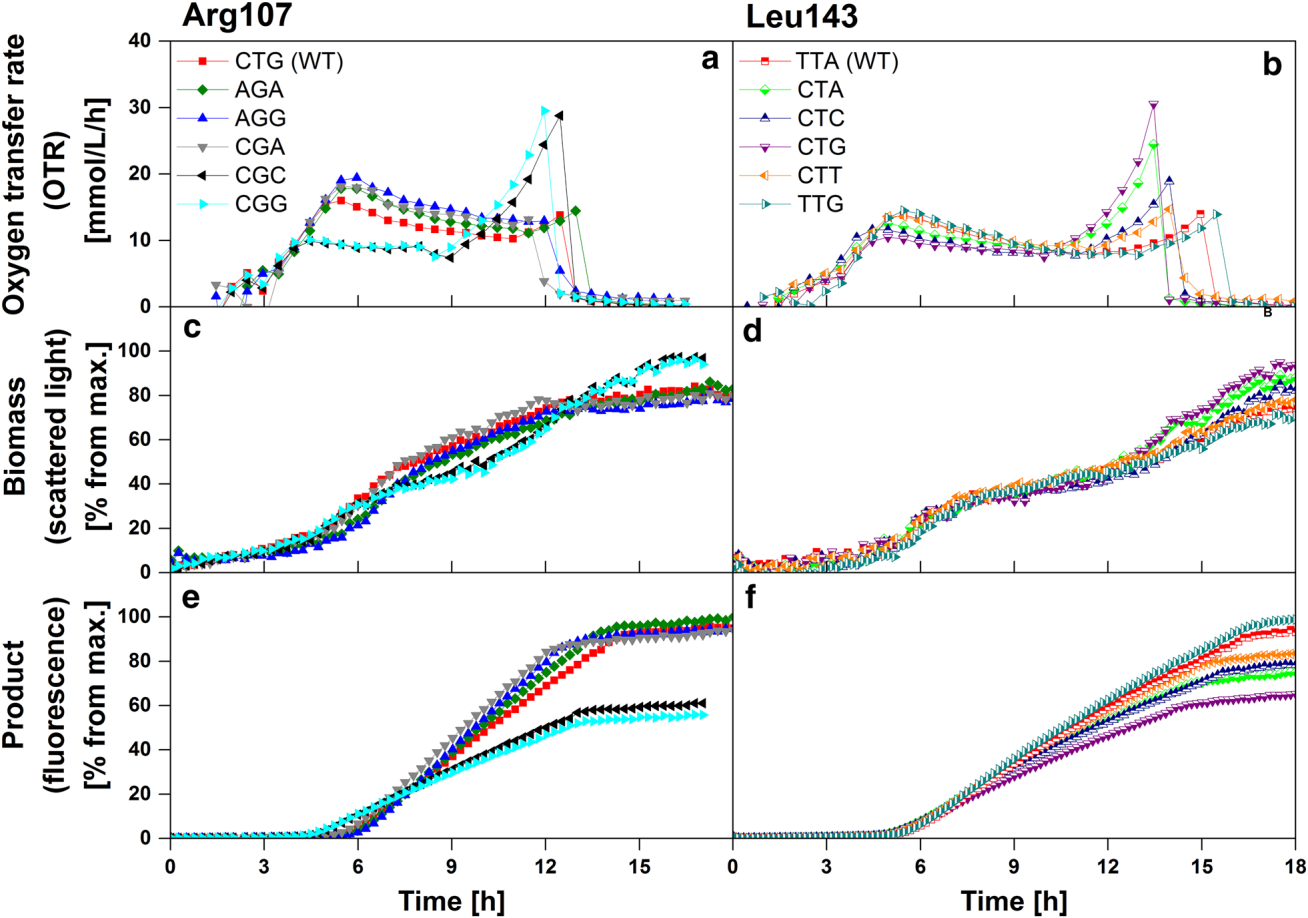
Respiration activity, biomass and product formation of 12 *E. coli* BL21(DE3) clones expressing BSLA under inducing conditions. Oxygen transfer rate (**a**,** b** determined using a RAMOS device), biomass (**c**,** d**) and product formation (**e**,** f** both determined using a BioLector device) as function of time for 12 *E.* *coli* BL21(DE3) clones cultivated in Wilms-MOPS mineral autoinduction medium containing 0.5 g/L glucose, 5 g/L glycerol, 2 g/L lactose (**a**,** c**,** e** six clones varying in Arg107 codons;** b**,** d**,** f** six clones varying in Leu143 codons). Cultivation conditions: 37 °C, 250 mL flasks, filling volume 10 mL, shaking frequency 350 rpm, shaking diameter 50 mm (in RAMOS); 37 °C, 48-well Flowerplate, filling volume 1 mL, shaking frequency 1000 rpm, shaking diameter 3 mm (in BioLector)

To determine the impact of silent codon exchanges in the heterologous gene on respiration activity, biomass and product formation during recombinant protein production under inducing cultivation conditions, main cultivations were performed in Wilms-MOPS mineral autoinduction medium containing lactose as inducing compound. Figure 2 presents OTR, biomass (scattered light intensity), and product formation (fluorescence signal) as function of time for all investigated clones varying in either Arg107 or Leu143 codons. To allow a detailed analysis of the differences among the clones, in all cultivations oxygen-unlimited conditions were guaranteed. Thereby, an overlaying effect of oxygen limitation on the metabolic activity of the clones [23] can be prevented.

Within the first hours of cultivation, growth on the preferred carbon source glucose which represses production leads to an increase in the OTR of all clones (Fig. 2a, b). With glucose depletion after roughly 3 h, the OTR increase is shortly interrupted. The following progress of the OTR strongly varies between the clones. For some clones (e.g. Arg107-CGC, -CGG, Leu143-CTA, -CTG) the OTR increases to 10 mmol/L/h after 4–5 h, then remains constant for 5–6 h. This is probably due to metabolic burden during recombinant protein production [22, 23]. Afterwards, the OTR value strongly increases up to a maximum of 25–30 mmol/L/h before dropping to 0 mmol/L/h at the end of the cultivation. In contrast, the OTR of other clones [e.g. Arg107-CTG (WT), -AGA, Leu143-TTA (WT), -TTG] first increases to 15–20 mmol/L/h after 5 h, then slightly decreases for about 6–7 h, before either directly dropping to 0 mmol/L/h or slightly increasing to about 10–15 mmol/L/h before dropping to 0 mmol/L/h. Slight differences within the lag phase and, in consequence, the total cultivation duration of both wild type clones [Arg107 CTG (WT) and Leu143 TTA (WT)] can be attributed to smallest differences within the progress of the precultivations, as well as the half-hourly OTR measuring cycle. Whereas the investigated clones differing in synonymous arginine codons depict two distinct OTR profiles, the clones differing in leucine codons show a continuous change in OTR profiles. As already described by Rahmen et al. [23] and other former studies [21, 22], a classification of the clones into two groups (Type A and Type B) according to their OTR profiles was undertaken to allow a simplified discussion about general differences observed. As presented in Additional file 1 (adapted from Rahmen et al. [23]), this classification is based on the ratio between integral X (from first OTR peak to local minimum) and integral Y (from minimum to second peak). Clones characterized by a ratio X/Y >1.2 can be categorized into Type A, clones with a ratio X/Y <1.2 into Type B [23]. Calculated mean X/Y ratios of all cultivations of the investigated clones, standard deviations and the resulting classifications are given in Table 1. The generally low standard deviation (maximum 1.6 for Arg107-AGA) also emphasizes the high reproducibility of the OTR profiles of all clones.

**Table 1:** Investigated codon exchanges in a wild type *Bacillus subtilis* lipase A protein, codon usage, X/Y ratio, and classification

Position within amino acid sequence	Investigated nucleotide triplet	Investigated codon	Codon usage in *E.* *coli* ^a^	Recognized by tRNA	Relative tRNA content in *E. coli* ^b^	Arithmetic mean X/Y ratio	Standard deviation^c^	Classification into Type A or Type B^d^
Arg107	CGT (WT)	CGU (WT)	0.42	Arg: 1, 2	0.90	2.0	0.2 (8)	A
Arg107	AGA	AGA	0.04	Arg: AGR	Minor	6.2	1.6 (3)	A
Arg107	AGG	AGG	0.03	Arg: AGR	Minor	8.8	1.1 (4)	A
Arg107	CGA	CGA	0.05	Arg: 1, 2	0.90	13.4	1.1 (3)	A
Arg107	CGC	CGC	0.37	Arg: 1, 2	0.90	0.8	0.2 (4)	B
Arg107	CGG	CGG	0.08	Arg: CGG	Minor	0.7	0.0 (4)	B
Leu143	TTA (WT)	UUA (WT)	0.11	Leu: UUR	0.25	2.0	0.2 (8)	A
Leu143	CTA	CUA	0.03	Leu: CUA	Minor	1.1	0.1 (4)	B
Leu143	CTC	CUC	0.10	Leu: 2	0.30	1.9	0.2 (4)	A
Leu143	CTG	CUG	0.55	Leu: 1	1.00	0.9	0.0 (3)	B
Leu143	CTT	CUU	0.10	Leu: 2	0.30	2.4	0.5 (4)	A
Leu143	TTG	UUG	0.11	Leu: UUR	0.25	3.1	0.2 (4)	A

*E. coli* BL21(DE3) clones containing plasmid pET22b(+) harboring the gene encoding His6–LOV–BSLA with wild type BSLA sequence composed of 181 amino acids. The wild type nucleotide triplets at positions 107 (arginine; Arg107) and 143 (leucine; Leu143) were changed into all possible synonymous triplets (1st and 2nd column). The investigated *E. coli* clones were named according to the nucleotide triplets within the gene. For all respective codons within the mRNA of *E. coli* (3rd column) the codon usage is given [44, 45] (4th column). For all codons the respective recognizing tRNA (5th column) and its relative contents in *E. coli* (6th column) are presented [39, 49]. The arithmetic mean of the X/Y ratio (Additional file 1) as well as the standard deviation according to Rahmen et al. [23] were determined for all individual cultivations of all clones (7th and 8th column). According to the critical X/Y ratio [23], all investigated clones are classified into Type A or Type B group (9th column)

^a^Abundance of respective codon relative to all codons for particular amino acid [44, 45]

^b^Taken from [39, 49]; the content is the relative amount to that of tRNALeu:1(CUG) that is normalized to 1.0 and approximately on the order of 10^4^ molecules per cell for normally growing *E. coli* [49]

^c^In brackets: number of individual cultivations used for calculation of arithmetic mean X/Y ratio and standard deviation

^d^Corresponding to critical X/Y ratio [23]

Beside clones varying in silent codon exchanges, also clones containing the amino acid exchanges S56P, D91R, G93Y, S167P, K170E, and G175F each encoded by at least two different synonymous codons were investigated. These additional mutations were included to demonstrate that the effect can also be observed at positions other than the two investigated in great detail with all possible codons. For the additional codon exchanges, the same OTR patterns were observed and a classification into Type A and Type B was also possible for those clones (Additional file 2). Thus, the phenomena observed in this work are independent from the position of the introduced mutations and emphasize our conclusions.

Figure 2c–f present the biomass and product formation of the investigated clones. Tremendous differences are obvious. Especially clones varying in leucine codons with a continuous change in OTR profiles illustrate the underlying relation between OTR, biomass and product formation: the more the clones behave according to Type A respiration behavior (longer time of active respiration, lower final OTR peak, higher X/Y ratio), the lower is the final biomass (scattered light intensity) and the higher the product formation (fluorescence signal). For clones varying in arginine codons, this correlation is less pronounced due to their very similar respiration behavior (OTR) within either Type A or Type B group. These clones show very distinct OTR profiles of Type A and Type B. Generally, all clones categorized into Type A result in lower final biomass and higher product formation. In contrast, Type B clones reach higher final biomass and show lower product formation. In addition to OTR, also biomass and product formation indicate high reproducibility. The maximum standard deviations of triplicate measurements for biomass and product are 3.56 and 2.53 %, respectively.

### Codon usage and tRNA abundance

Several studies already discussed the influence of rare codons on recombinant protein production [19, 34–37], as well as the molecular background [35, 38, 39] and strategies to overcome codon bias [35, 40–43]. As nicely presented by Gustafsson et al., the average codon preferences of *E. coli* and *Bacillus* (original host of the target gene *lipA* encoding BSLA) are quite similar since both organisms cluster in principal component analysis [35]. Therefore, a profound impact of codon bias on heterologous gene expression was not expected in this study, in particular, since only a single codon was replaced by a synonymous one. Nevertheless, the codon usage of *E. coli* [44, 45] for all synonymous Arg107 and Leu143 codons was analyzed with the aim to investigate possible influencing factors provoking the two types of respiration behavior (Table 1). As a result, the codon usage neither affected nor caused the classification of the clones into Type A and Type B respiration behavior. This can easily be recognized by comparing the codon usage of Type B clones (grey background in Table 1). For Arg107 as well as Leu143, one codon each has a very high frequency, whereby the usage of the other respective synonymous codon is quite low.

Since the tRNA concentration is known to influence the translation efficiency and, thereby, recombinant protein production [38, 46–48], also the relative tRNA content in *E. coli* [39, 49] was taken into account. For the investigated arginine and leucine codons, the abundance of the respective tRNAs can roughly be correlated with the abundance of the recognized codons (Table 1). Thus, the differences in tRNA content are not responsible for the observed Type-A–Type-B-classification. To definitely exclude a tRNA limitation as influencing factor, in future a more detailed tRNA analysis over the cultivation time is necessary since the tRNA level may vary with cell density. Nevertheless, in this study a tRNA limitation is not expected since only a single codon within the heterologous *lipA* gene was substituted.

### Nucleobase analysis

**Fig. 3:**
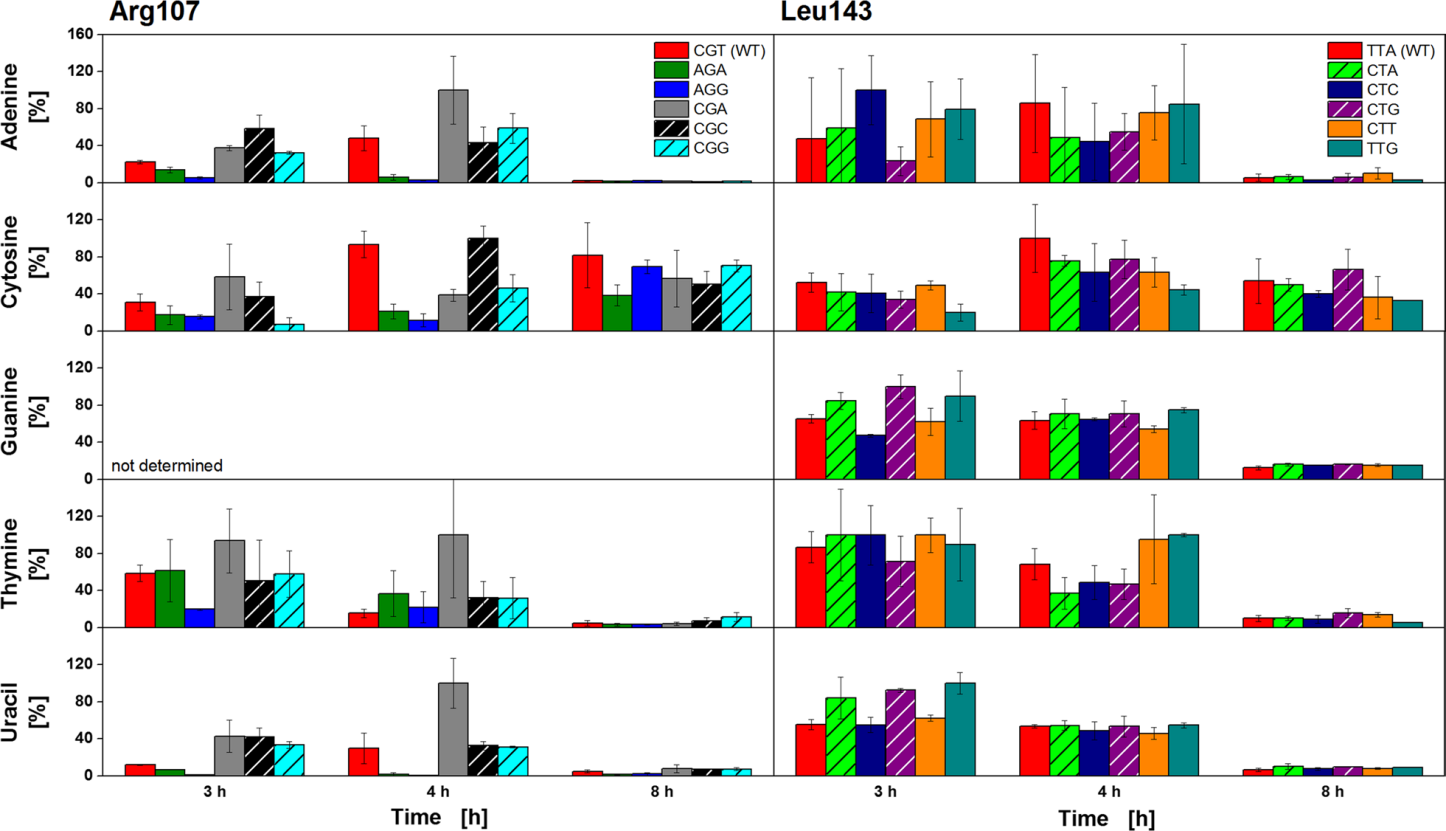
Relative changes in intracellular nucleobases during initial growth and production phases. Time-dependent changes in free nucleobases within the cells presented for six clones varying in Arg107 codons (*left*) and six clones varying in Leu143 codons (*right*) as relative intensities in [%]. At the indicated time points, the cells were collected, their metabolites extracted, and the nucleobases were analyzed by LC/MS. Clones categorized into Type A respiration behavior are presented with *fully filled bars*, Type B clones are indicated with *striped bars*. The three chosen time points represent initial growth on glucose (3 h) and glycerol (4 h), and recombinant protein production on lactose (8 h). All data points are the mean of triplicate measurements. The *error bars* represent the standard deviation

As a further factor possibly influencing respiration and causing the two observed types of respiration behavior, the availability of free nucleobases in the cells was investigated since a nucleobase or nucleotide limitation might contribute to metabolic burden. Figure 3 presents relative intensities of free intracellular nucleobases at three different time points of the cultivation. The relative intensities were calculated according to the maximum value for each nucleobase after division by the intensity of the internal standard. As will be discussed later, the three time points represent initial growth on glucose (3 h) and glycerol (4 h), as well as recombinant protein production on lactose (8 h). They were chosen to represent the time points of differentiation between Type A and Type B clones within the initial growth and protein production phases.

Generally, the relative intensities of all nucleobases found in the investigated *E. coli* clones show quite high standard deviations, probably due to a complicated sample preparation and, therefore, impede a detailed analysis of the results. During the protein production phase (8 h), the intracellular nucleobases adenine, guanine, thymine, and uracil are only present in quite low relative intensities compared to those at earlier time points due to the increased demand in nucleobases during plasmid replication, transcription and translation process within protein production. In contrast, the relative cytosine intensity is roughly constant compared to intensities measured during growth on glucose (3 h) and glycerol (4 h). The rather small reduction of intracellular cytosine during recombinant protein production could be attributed to its lower occurrence (19.9 %) within the *lipA* gene sequence compared to adenine (30.2 %), guanine (26.3 %) and thymine (23.6 %). Despite these general findings, no correlation concerning the classification into Type A and Type B respiration behavior was found. No significant differences between the relative intracellular nucleobase intensities of Type A (Fig. 3, fully filled bars) and B clones (Fig. 3, striped bars) were observed. In conclusion, a nucleobase limitation can be excluded as reason for the two observed types of respiration behavior. For Type-A–Type-B-classification, a large number of other metabolites was also analyzed (Additional file 6.5: Table). However, no correlation was yet identified.

### In-depth characterization of quantitative cultivation parameters

**Fig. 4:**
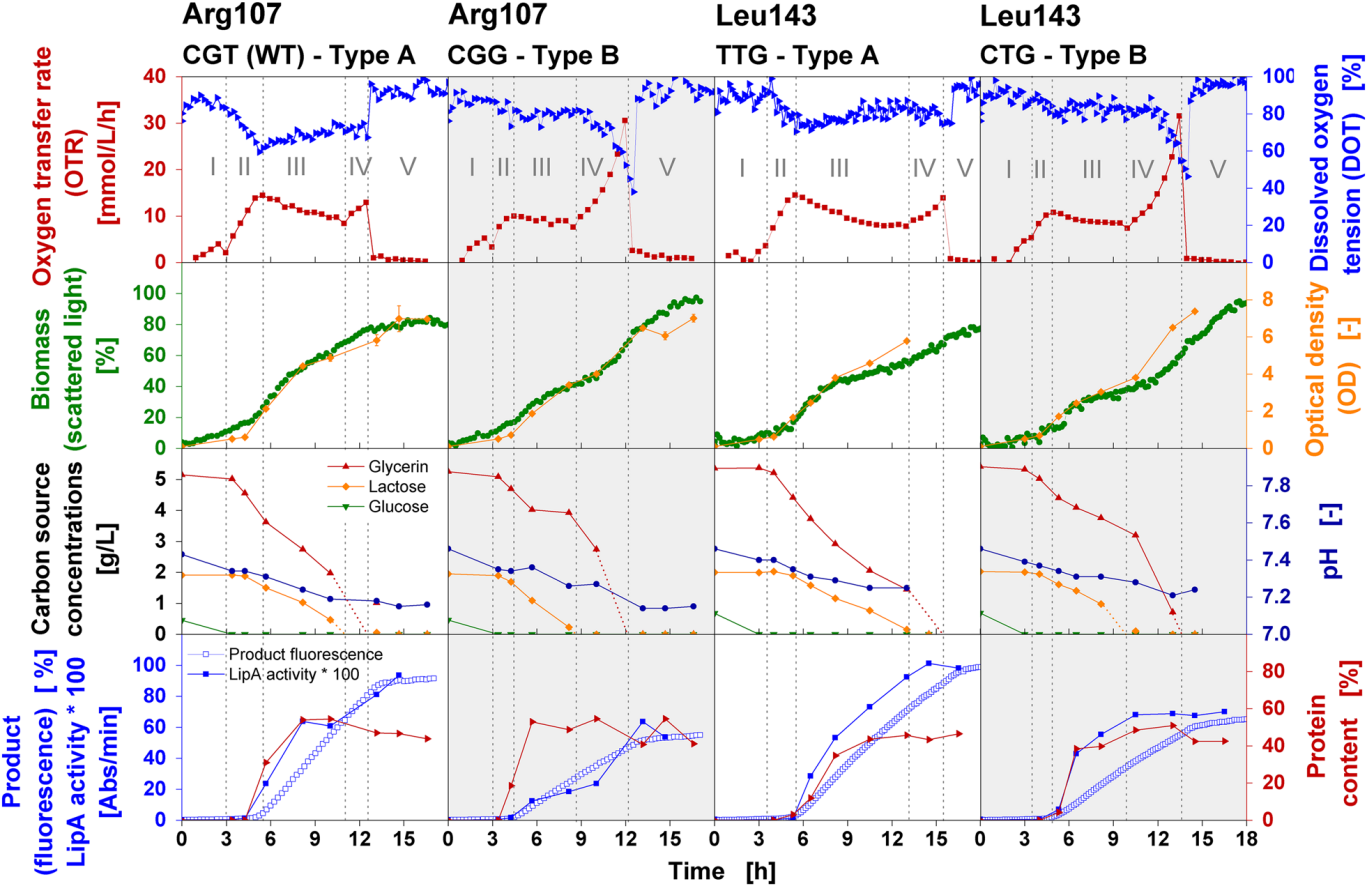
Cultivation parameters of four representative *E. coli* BL21(DE3) clones of Type A and Type B behavior expressing BSLA under inducing conditions. Characterization of four *E.* *coli* BL21(DE3) clones belonging to respiration behavior Type A [Arg107-CGT (WT), Leu143-TTG; *white background*] or respiration behavior Type B (Arg107-CGG, Leu143-CTG; *grey background*) during cultivation in Wilms-MOPS mineral autoinduction medium containing 0.5 g/L glucose, 5 g/L glycerol, 2 g/L lactose. Cultivation parameters: oxygen transfer rate (OTR, determined using a RAMOS device), biomass (scattered light), soluble product formation (fluorescence), dissolved oxygen tension (DOT, all determined using a BioLector device), carbon source concentrations, optical density (OD), pH-value, enzyme activity and protein content of the target protein per total protein of the cell (all determined from parallel experiments in conventional shake flasks). The *red* and *orange dotted lines* represent the expected depletion of glycerol and lactose, respectively, based on the OTR profile. The *vertical grey dotted lines* separate the five cultivation phases (I–V) identified by the OTR curves according to Rahmen et al. [23]. Cultivation conditions: 37 °C, 250 mL flasks, filling volume 10 mL, shaking frequency 350 rpm, shaking diameter 50 mm (in RAMOS and conventional flasks); 37 °C, 48-well Flowerplate, filling volume 1 mL, shaking frequency 1000 rpm, shaking diameter 3 mm (in BioLector)

For in-depth characterization of cultivation parameters, *E. coli* clones differing only in a particular silent codon exchange were cultivated and the online signals OTR (using a RAMOS device), biomass (scattered light intensity), product formation (fluorescence signal), and dissolved oxygen tension (DOT, all using a BioLector device) were determined. Furthermore, carbon source concentrations, optical density (OD), pH-value and biomass-specific target protein content were determined from offline samples (collected from parallel experiments in conventional shake flasks). For one representative Type A and Type B clone varying in either Arg107 or Leu143 codon the cultivation parameters are presented as a function of time (Fig. 4). For all further clones, the respective parameters are presented in Additional file 3.

According to our previous findings [23], the cultivation can be separated into five characteristic cultivation phases: (I) growth on glucose, (II) growth on glycerol, (III) induction of gene expression and recombinant protein production on lactose, (IV) growth on residual glycerol, (V) end of cultivation. As presented in Fig. 4, during glucose consumption (I), OTR, biomass (scattered light intensity) and OD slightly increase and no product formation is observed. During growth on glycerol (II), biomass and OD further increase. The OTR further increases until the slope of the curve has decreased to 0. Apparently, the metabolism gradually switches from only glycerol to mixed glycerol/lactose consumption and some initial target protein is produced. The duration of this phase (II) is shorter for Type B (1.5 h) compared to Type A clones (2–2.5 h) and, therefore, the biomass obtained within this phase is slightly lower for Type B (OD ≤ 1) than Type A clones (OD ≥ 2). During the third cultivation phase (III), strong protein formation is observed, the increase in biomass and OD is reduced, and the OTR slightly decreases or stays roughly constant. The biomass-specific protein content represents the relative amount of recombinant lipase with respect to total protein content. Product fluorescence signal and enzyme activity are both indicators for soluble and correctly folded protein per culture volume. To better determine between both protein fractions, Additional file 4 presents SDS page analysis for soluble and insoluble fractions for the four clones. Whereas Type A clones metabolize lactose and glycerol in parallel, Type B clones consume mainly lactose. The duration of this phase is slightly longer for Type A (6–8 h) than Type B clones (4–5 h). With depletion of lactose, the third phase is terminated and the maximum biomass-specific protein content is reached (roughly 50 % of total protein). During the fourth phase (IV), residual glycerol is consumed leading to another increase in OTR, biomass, and OD which is much stronger for Type B than Type A clones due to the higher residual glycerol concentration (3–3.5 g/L for Type B compared to 1–1.5 g/L for Type A clones). With glycerol depletion, the OTR typically drops at the end of the cultivation (V). During the entire cultivation, the pH-value only slightly decreases from 7.4 to 7.2 due to the well-buffered medium. For all clones, the DOT mirrors the OTR indicating coinciding cultivation conditions in shake flasks and microtiter plate. This has also been found in a recent publication with different microorganisms in well-adjusted shake flask and microtiter plate cultivation experiments [50]. Type A clones reach higher enzyme activities (0.8–0.9 Abs/min) and higher final fluorescence signals (90–100 %) representing soluble protein compared to Type B (0.5–0.6 Abs/min, 60–70 %), while final biomass (scattered light intensity) is lower (65–75 %) compared to Type B clones (90–100 %). Thus, the host cell metabolic burden correlates with recombinant protein production. Kunze et al. [22] found in their investigations a strong influence on the metabolic activity upon expression of different human proteins. Furthermore, the same phenomenon observed here for the lipase (BSLA) could also be found for an oxidoreductase from the thermophilic bacterium *Thermus thermophilus* [51]. Therefore, a probable negative effect of the lipase activity is not assumed in this study. An effect of a possibly limiting flavin incorporation on the differences between Type A and Type B behavior can be excluded since both signals representing the soluble and correctly folded protein—fluorescence signal and enzyme activity—correlate.

In conclusion, major differences between Type A and Type B clones consist of different durations of the second and third cultivation phase as well as the altered kinetics of the glycerol and lactose consumption. The shorter phase (II) of Type B clones results in a slightly reduced biomass at the beginning of next cultivation phase. During phase (III), which is characterized by strong protein production, Type B clones predominantly consume lactose. As consequence, on the one hand, lactose is depleted earlier leading to the shortened duration of phase (III), on the other hand, a high residual glycerol concentration is available for consumption during cultivation phase (IV), leading to the characteristic sharp OTR increase of Type B clones. All observed differences between Type A and Type B clones are very consistent and are of phenomenological nature and make further detailed analysis necessary to gain a deeper understanding of the underlying molecular mechanisms.

### Plasmid copy number and mRNA analysis

**Fig. 5:**
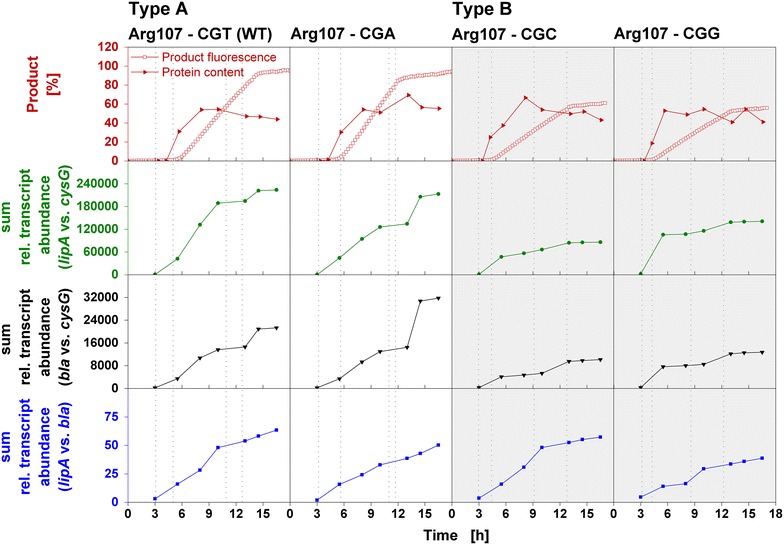
Product formation and sum of relative transcript abundances of four *E. coli* BL21(DE3) clones grown under inducing conditions. Soluble and total product formation (determined using a BioLector device or densitometric analysis, respectively) and sum of relative transcript abundances (determined from conventional shake flasks) as function of time for four *E.* *coli* BL21(DE3) clones belonging to respiration behavior Type A [Arg107-CGT (WT), -CGA; *white background*] or respiration behavior Type B (Arg107-CGC, -CGG; *grey background*) during cultivation in Wilms-MOPS mineral autoinduction medium containing 0.5 g/L glucose, 5 g/L glycerol, 2 g/L lactose. ‘*lipA* vs. *cysG*’ represents the sum of the relative transcript abundance of the plasmid-encoded target gene *lipA* (encoding *Bacillus subtilis* lipase A) in relation to the genome-encoded reference gene *cysG* (encoding uroporphyrin III C-methyltransferase). ‘*bla* vs. *cysG*’ represents the relative plasmid copy number as the sum of the relative transcript abundance plasmid-encoded reference gene *bla* (encoding beta-lactamase) in relation to the genome-encoded reference gene *cysG.* ‘*lipA* vs. *bla*’ represents the factor of *lipA* induction as transcript abundance of the plasmid-encoded target gene *lipA* in relation to the plasmid-encoded reference gene *bla*. The *grey dotted lines* separate the five cultivation phases presented in Fig. 4. Cultivation conditions: 37 °C, 250 mL flasks, filling volume 10 mL, shaking frequency 350 rpm, shaking diameter 50 mm (in conventional flasks); 37 °C, 48-well Flowerplate, filling volume 1 mL, shaking frequency 1000 rpm, shaking diameter 3 mm (in BioLector)

Previous results hinted at an influence of the plasmid copy number on respiration behavior [23].Therefore, plasmid copy number as well as mRNA content were analyzed in this study. As presented in Fig. 5, for two Type A and two Type B clones varying in Arg107 codons, the online-determined formation of soluble product (fluorescence signal), total recombinant protein content and the sum of relative transcript abundances were determined. The results are based on the expression of the plasmid-encoded target gene *lipA* (encoding *B. subtilis* lipase A), the genome-encoded reference gene *cysG* (encoding uroporphyrin III C-methyltransferase), and the plasmid-encoded reference gene *bla* (encoding beta-lactamase). ‘*lipA* vs. *cysG*’ represents the expression level of *lipA* relative to *cysG*, ‘*bla* vs. *cysG*’ represents the relative expression level of plasmid-encoded *bla* and indicates the plasmid copy number, ‘*lipA* vs. *bla*’ represents the induced *lipA* expression relative to basal plasmid expression and indicates the overexpression factor. The respective data for four clones varying in Leu143 codons is presented in Additional file 5.

In general, the product formation (fluorescence signal) as well as the sum of the relative transcript abundances ‘*lipA* vs. *cysG*’ and ‘*bla* vs. *cysG*’ show a concordant progress over time: an increase from the time point of induction (4–5 h) until the end of cultivation (12–15 h). The average standard deviation of the best two out of three technical replicates of all determined transcript abundances is 3.29 %, the maximum standard deviation 12.40 %. The factor of overexpression (‘*lipA* vs. *bla*’) is roughly the same for the investigated clones. In contrast, Type A clones [Arg107-CGT (WT) and -CGA, white background] reach considerably higher final values for product (fluorescence signal), as well as for the cumulative transcript abundances of *lipA* and *bla* relative to *cysG* compared to Type B clones (Arg107-CGC and -CGG, grey background).

These results indicate strong differences between Type A and Type B clones. The plasmid copy number and, in consequence, mRNA level and product formation are clearly elevated for Type A clones. These variations probably result from the different biomass of Type A and Type B clones at the beginning of the protein production phase (III, Fig. 4). At this time point of cultivation, the biomass is higher for Type A than for Type B clones. The subsequent induction of gene expression results in excessive plasmid replication and, consequently, in an increase in plasmid copy number as previously described in literature [18, 23] and also observed in this study. This greatly contributes to the host cell metabolic burden [1, 16–18]. As a result, the metabolic activity is strongly impaired during the phase of gene expression (III) indicated by the decelerated increase in biomass (scattered light intensity) and optical density, as well as the constant or decreasing OTR within the induction phase (III, Fig. 4). Due to the higher plasmid copy number of Type A clones, the host cell metabolism is even more burdened, resulting in lower final biomasses (scattered light intensities) and only slight OTR increases at the end of the cultivation.

In addition to the mRNA content also its secondary structure can affect recombinant protein production since it may influence the translation elongation rate. Whereas strong secondary structures containing mRNA hairpins lead to slower translation, faster translation is possible for mRNA molecules with weaker secondary structures [52]. In this study, no change in secondary structure is expected since only a single codon within the target gene was replaced by a synonymous one. Nevertheless, in future, the *lipA* gene expression should be analyzed using a dynamic model of prokaryotic gene expression referring to gene sequence information. Therewith, the impact of small modifications in the *lipA* gene sequence on the dynamics of expression rates could mechanistically be assessed and sequence-related effects on the stages of mRNA synthesis, mRNA degradation and ribosomal translation could be taken into account [53].

In this study, not only differences on mRNA and protein level between Type A and Type B were observed, but also differences in plasmid copy number. Thus, in addition to transcription and translation also plasmid replication should be further evaluated in future. Furthermore, the molecular rationales for the differences in biomass at the beginning of the induction phase that probably lead to the observed differences in plasmid copy number are of major interest and require further analysis.

## Conclusions

In this study, the influence of particular silent codon exchanges in a heterologous *lipA* gene encoding the *Bacillus subtilis* lipase A on metabolic activity and recombinant protein production of *E. coli* BL21(DE3) was investigated. Therefore, *E. coli* clones differing in synonymous arginine or leucine codons as well as clones containing the amino acid exchanges S56P, D91R, G93Y, S167P, K170E, and G175F each encoded by different synonymous codons were compared during cultivation under inducing conditions. These smallest possible variations in the heterologous gene led to significant and highly reproducible differences in respiration activity, biomass and protein production. According to their respiration behavior the clones could be classified into two groups, Type A and Type B, respectively. Whereby the group of Type A clones indicated higher product formation, Type B clones were identified as clones with stronger biomass formation. The clones were further analyzed for potential factors affecting the observed differences. Intracellular nucleobases and codon usage neither showed any impact on both groups nor did they cause the differences between Type A and Type B clones. In contrast, Type B clones exhibited a reduced initial growth resulting in a lower biomass concentration at the time point of induction. As a consequence, plasmid copy numbers and expression levels remained lower, and the kinetics of the carbon source (lactose and glycerol) consumption changed. Since these phenomena were observed for all investigated clones, our findings do not depend on the specific encoded amino acid or its position and, thus, are of general nature.

A silent codon exchange within the *lipA* gene represents the only difference between the clones investigated here. Thus, all phenomenological can be attributed to these small differences. In conclusion, this study demonstrates that a particular silent codon exchange in a heterologous gene sequence tremendously affects host cell metabolism and recombinant protein production during cultivation under inducing conditions. Although the rationales and molecular mechanisms responsible for the observed effects are not yet understood, these findings are highly significant for codon optimization of heterologous genes, screening procedures for improved variants, and biotechnological protein production processes.

## Methods

### Microorganism and target protein

*Escherichia coli* DH5α was used for cloning and amplification. All cultivation experiments were conducted with *E. coli* BL21(DE3) containing plasmid pET22b(+) (Novagen, Merck, Germany) harboring the gene encoding the fusion protein His_6_–LOV–BSLA composed of a N-terminal His_6_ tag (His_6_), a flavin-based fluorescent protein derived from the light, oxygen, voltage domain of the *Bacillus subtilis* YtvA photoreceptor (LOV) [25, 26], and a wild type *Bacillus subtilis* lipase A (BSLA) composed of 181 amino acids [24]. The investigated *E. coli* clones carry silent codon exchanges at position 107 (arginine, Arg107) and position 143 (leucine, Leu143) within the BSLA sequence. The wild type codons were changed into all possible synonymous codons (Table 1). An additional set of variants carries synonymous codon exchanges for the amino acid substitutions S56P, D91R, G93Y, S167P, K170E, and G175F.

### Site-directed mutagenesis

Site-directed silent mutagenesis was applied to introduce silent mutations of *lipA* gene encoding BSLA. Polymerase chain reactions (PCR) were executed using the modified SPRINP method of Edelheit et al. [54]. All reaction conditions were already described by Rahmen et al. [23] and are summarized in Additional file 6.1. Successful mutagenesis was guaranteed by sequencing (Eurofins MWG Operon, Germany). Constructed plasmids carrying the silent mutations within BSLA were transformed into chemically competent *E.coli* BL21(DE3) cells and preserved in 15 % (w/w) glycerol at −80 °C.

### Cultivation media

Two non-inducing and one autoinduction medium were used as cultivation media. The first precultivation was performed in non-inducing complex Terrific Broth (TB) [55] medium. The second precultivation was carried out in non-inducing modified Wilms-MOPS mineral medium according to Wilms et al. [56] containing 5 g/L glycerol and 0.5 g/L glucose as carbon sources. For growth under inducing conditions, the modified Wilms-MOPS mineral medium was supplemented with 2 g/L sterile lactose as inducing compound [5, 6, 57]. This medium is referred to as Wilms-MOPS mineral autoinduction medium. The detailed media composition is presented in Additional file 6.2.

### Cultivations and online analysis using RAMOS and BioLector devices

All cultivations were carried out according to Rahmen et al. [23]. Precultivations were performed in modified 250 mL shake flasks within a self-made RAMOS device [27, 28]. Commercial versions of the RAMOS device are available from Kuhner AG, Switzerland or HiTec Zang GmbH, Germany. Main cultivations were conducted in three cultivation systems in parallel: (I) modified shake flasks in RAMOS [27, 28] to online determine the oxygen transfer rate (OTR) as indicative parameter for growth and metabolic activity, (II) conventional 250 mL shake flasks to allow offline sample analysis of various cultivation parameters, (III) 48-well Flowerplate (m2p-labs GmbH, Germany) in a self-constructed BioLector device [29, 30] to online determine volumetric biomass and product formation as well as dissolved oxygen tension (DOT). Commercial versions of the BioLector device are available from m2p-labs GmbH, Germany. Cultivation conditions were as follows: 37 °C, 250 mL flasks, filling volume 10 mL, shaking frequency 350 rpm, shaking diameter 50 mm (in RAMOS and conventional flasks); 37 °C, 48-well Flowerplate, filling volume 1 mL, shaking frequency 1000 rpm, shaking diameter 3 mm (in BioLector). The cultivation conditions were chosen to guarantee oxygen-unlimited conditions. The first preculture was inoculated with 100 µL from a stock culture and harvested after 3 h at OTR of 35–50 mmol/L/h. The second preculture was inoculated with culture broth from the first precultivation with an initial optical density (OD) set at 0.1 and harvested after 5 h at OTR of 28–33 mmol/L/h. For main cultivations, a master mix was inoculated with culture broth from the second preculture with initial OD set at 0.1.

### Offline analysis of cultivation parameter

#### Carbon sources

Carbon source concentrations of glucose, lactose, and glycerol were analyzed by HPLC. Conditions are presented by Rahmen et al. [23] and summarized in Additional file 6.3.

#### Recombinant protein

Recombinant target protein (BSLA) based on biomass was determined via sodium dodecylsulfate polyacrylamide gel electrophoresis (SDS-PAGE) and subsequent densitometry. A detailed description is given by Rahmen et al. [23] and in Additional file 6.4.

#### Optical density

Optical density (OD) was determined at wavelength 600 nm in 1 cm cuvettes in a spectrophotometer (Genesys 20, Thermo Scientific, Germany) in duplicates. To keep OD in the linear range between 0.1 and 0.5, samples were diluted with fresh medium. Fresh medium was used as blank.

#### pH-value

The pH-value was determined by InLab Easy pH electrode (Mettler Toledo, Germany) with CyberScan pH 510 meter (Eutech Instruments, Thermo Scientific, Germany).

#### Sampling, metabolite extraction and metabolome analysis

Sample preparation and metabolite extraction were performed according to Izumi et al. [58]. For sample preparation, culture broth was filtered under vacuum suction. Filter-bound cells were frozen in liquid nitrogen to quench metabolism. Cell metabolites were extracted using methanol–water–chloroform (5:2:2) extraction, polar and nonpolar phase metabolites were separated and finally lyophilized before analysis. A detailed description is given in [58] and Additional file 6.5. Cell extracts were analyzed according to Huang et al. [59] by (1) pentafluorophenylpropyl (PFPP) stationary phase liquid chromatography (Discovery HS F5, 150 mm × 2.1 mm, particle size 3 µm, Sigma-Aldrich Corp., Germany) coupled with electrospray ionization (ESI) in positive and negative modes; and (2) reversed phase ion pairing liquid chromatography with a C18 column (CERI L-column 2 ODS, 150 mm × 2.1 mm, particle size 3 μm, Chemicals Evaluation and Research Institute, Kyoto, Japan) coupled with ESI in negative mode, to a triple-quadrupole mass spectrometer (LCMS 8030 plus; Shimadzu, Japan). A detailed description of mobile phases, concentration gradients, flow rates, injection volumes, column oven temperatures, as well as MS parameters is given in Additional file 6.5.

#### RNA extraction and quantification of transcript abundance

Total RNA was purified from *E.coli* cells grown in Wilms-MOPS mineral autoinduction medium. Cells were harvested from 1 mL of the suspension culture after centrifugation using a Sigma 1–15 K centrifuge at 21,918*g*. RNA was extracted from bacterial cells using peqGOLD RNA Pure (Peqlab, Germany) according to the manufacturer’s instructions and RNA concentration was determined with a NanoPhotometer P330 (Implen, Germany). Relative transcript abundance was determined via reverse-transcription quantitative PCR (RT-qPCR) performed on an ABI 7300 Real-Time PCR System (Applied Biosystems, Life Technologies, Germany). Prior to reverse transcription, 1 μg RNA was treated with RNase-free DNaseI for digestion of contamination with genomic DNA. The cDNA was synthesized with RevertAid Reverse Transcriptase and random nonamer primers (Metabion GmbH, Germany). DNaseI and RevertAid Reverse Transcriptase were purchased from Thermo Fisher Scientific Bioscience GmbH, Germany. RT-qPCR was performed with gene specific primer sequences for *cysG*, *bla* and *lipA* (Table 2) designed with Primer3Plus [60] and synthesized by Eurofins MWG operon, Germany. The reference gene *cysG* was chosen based on its stable expression in recombinant over-expression studies [61]. Amplification was carried out using SYBR Green qPCR SuperMix-UDG with ROX (Invitrogen, Life Technologies, Germany) with cycling conditions as follows: Initial activation cycle at 50 °C for 2 min and denaturation at 95 °C for 10 min, 40 cycles of 15 s at 95 °C and 1 min at 60 °C. For determination of product specificity, a melt-curve analysis was performed and products were sequenced (Sequence Laboratories Göttingen GmbH, Germany). Relative transcript abundance of target genes relative to the reference gene was determined according to Livak and Schmittgen [62] using [2^(Ct(reference) − Ct(target))^].

**Table 2:** Primer sequences used for qPCR

Target	Primers (5′ → 3′)^a^	Length (nt)	Product size (bp)
*bla*	F: CCGGCGTCAATACGGGATAA	20	94
	R: TCCTTGAGAGTTTTCGCCCC	20	
*cysG*	F: GCTTCTGGTTGCTCTGCCTA	20	100
	R: GCTCGCCACCGGTTTTTAAG	20	
*lipA*	F: TTGACGACAGGCAAGGCG	18	92
	R: TTCATGACAATCATATCGGCACT	23	

^a^F and R indicate forward and reverse primers, respectively

## Additional files

10.1186/s12934-015-0348-8 Quantitative classification of E. coli BL21(DE3) clones according to respiration behavior adapted from Rahmen et al. [23]. Respiration behavior (oxygen transfer rate, determined using a RAMOS device) as function of time for one example each for a Type A and Type B clone. According to Rahmen et al. [23] integral X represents protein production phase, whereas integral Y represents the subsequent growth phase on glycerol. X/Y > 1.2 categorizes Type A clones, X/Y < 1.2 Type B clones. Calculated X/Y ratios for all investigated clones are given in Table 1. Cultivation conditions: 37 °C, 250 mL flasks, filling volume 10 mL, shaking frequency 350 rpm, shaking diameter 50 mm (in RAMOS).
10.1186/s12934-015-0348-8 Respiration activity of *E. coli* BL21(DE3) clones containing amino acid exchanges with synonymous codons grown under inducing conditions. Respiration behavior (oxygen transfer rate) as function of time for clones containing amino acid exchanges with synonymous codons during cultivation in Wilms-MOPS mineral autoinduction medium containing 0.5 g/L glucose, 5 g/L glycerol, 2 g/L lactose. Clones S56P, S167P, and G175F can be categorized into Type A independent from codon, clones K170E into Type B. Clones D91R and G93Y are categorized into Type A (D91R-CGC, G93Y-TAT) or Type B (D91R-CGT, G93Y-TAC) according to the respective synonymous codon. All cultivation experiments were performed at least in duplicates. Cultivation conditions: 37 °C, 250 mL flasks, filling volume 10 mL, shaking frequency 350 rpm, shaking diameter 50 mm (in RAMOS).
10.1186/s12934-015-0348-8 Cultivation parameters of eight *E. coli* BL21(DE3) clones grown under inducing conditions. Characterization of (A) four *E. coli* BL21(DE3) clones varying in Arg107 codon and (B) four clones varying in Leu143 codon, belonging to respiration behavior Type A (Arg107-AGA, -AGG, -CGA, and Leu143-CTC, -CTT, -TTA (WT); white background) or respiration behavior Type B (Arg107-CGC, Leu143-CTA; grey background) during the cultivation in Wilms-MOPS mineral autoinduction medium containing 0.5 g/L glucose, 5 g/L glycerol and 2 g/L lactose. Cultivation parameters: oxygen transfer rate (OTR, determined using a RAMOS device), biomass (scattered light), soluble product (fluorescence), dissolved oxygen tension (DOT, all determined using a BioLector device), carbon source concentrations, optical density (OD), pH-value, enzyme activity and protein content of the target protein per total protein of the cell (all determined from parallel experiments in conventional shake flasks). The red and orange dotted lines represent the expected depletion of glycerol and lactose, respectively, based on the OTR profile. The vertical grey dotted lines separate the five cultivation phases (I-V) identified by the OTR curves according to Rahmen et al. [23]. Cultivation conditions: 37 °C, 250 mL flasks, filling volume 10 mL, shaking frequency 350 rpm, shaking diameter 50 mm (in RAMOS and conventional flasks); 37 °C, 48-well Flowerplate, filling volume 1 mL, shaking frequency 1000 rpm, shaking diameter 3 mm (in BioLector).
10.1186/s12934-015-0348-8 SDS-PAGE analysis for soluble and insoluble protein fractions of four clones presented in Figure 4. SDS-PAGE analysis showing soluble (top) insoluble protein (bottom) per sample volume as function of time (target protein band is framed, M = protein marker) for the clones Arg107-CGT (WT, Type A), Arg107-CGG (Type B), Leu143-TTG (Type A) and Leu143-CTG (Type B).
10.1186/s12934-015-0348-8 Product formation and sum of relative transcript abundances of four *E. coli* BL21(DE3) clones grown under inducing conditions. Soluble product formation (determined using a BioLector device) and sum of relative transcript abundances (determined from conventional shake flasks) as function of time for four *E. coli* BL21(DE3) clones belonging to respiration behavior Type A (Leu143-CTT, -TTG; white background) and respiration behavior Type B (Leu143-CTA, -CTG; grey background) during cultivation in Wilms-MOPS mineral autoinduction medium containing 0.5 g/L glucose, 5 g/L glycerol, 2 g/L lactose. ‘lipA vs. cysG’ represents the sum of the relative transcript abundance of the plasmid-encoded target gene lipA (encoding Bacillus subtilis lipase A) in relation to the genome-encoded reference gene cysG (encoding uroporphyrin III C-methyltransferase). ‘bla vs. cysG’ represents the relative plasmid copy number as the sum of the relative transcript abundance plasmid-encoded reference gene bla (encoding beta-lactamase) in relation to the genome-encoded reference gene cysG. ‘lipA vs. bla’ represents the factor of lipA induction as transcript abundance of the plasmid-encoded target gene lipA in relation to the plasmid-encoded reference gene bla. Cultivation conditions: 37 °C, 250 mL flasks, filling volume 10 mL, shaking frequency 350 rpm, shaking diameter 50 mm (in conventional flasks); 37 °C, 48-well Flowerplate, filling volume 1 mL, shaking frequency 1000 rpm, shaking diameter 3 mm (in BioLector).
10.1186/s12934-015-0348-8 Supplement to Methods section.

## Abbreviations

*bla*: gene encoding beta-lactamase
BSLA: *Bacillus subtilis* lipase A
*cysG*: gene encoding uroporphyrin III C-methyltransferase
*E. coli*: *Escherichia coli*
ESI: electrospray ionization
FbFP: flavin-based fluorescent protein
HPLC: high performance liquid chromatography
IPTG: isopropyl *β*-d-1-thiogalactopyranoside
(LC)MS: (liquid chromatography) mass spectrometry
*lipA*: gene encoding BSLA
LOV: light, oxygen, voltage
OTR: oxygen transfer rate
PFPP: pentafluorophenylpropyl
RAMOS: respiration activity monitoring system
(RT-q)PCR: (reverse-transcription quantitative) polymerase chain reaction
SDS-PAGE: sodium dodecylsulfate polyacrylamide gel electrophoresis
