# Supplementary figures and images for: A particular silent codon exchange in a recombinant gene greatly influences host cell metabolic activity
Source: Microb Cell Fact. 2015 Oct 5;14:156. doi: 10.1186/s12934-015-0348-8 (PMC4595056; doi:10.1186/s12934-015-0348-8)

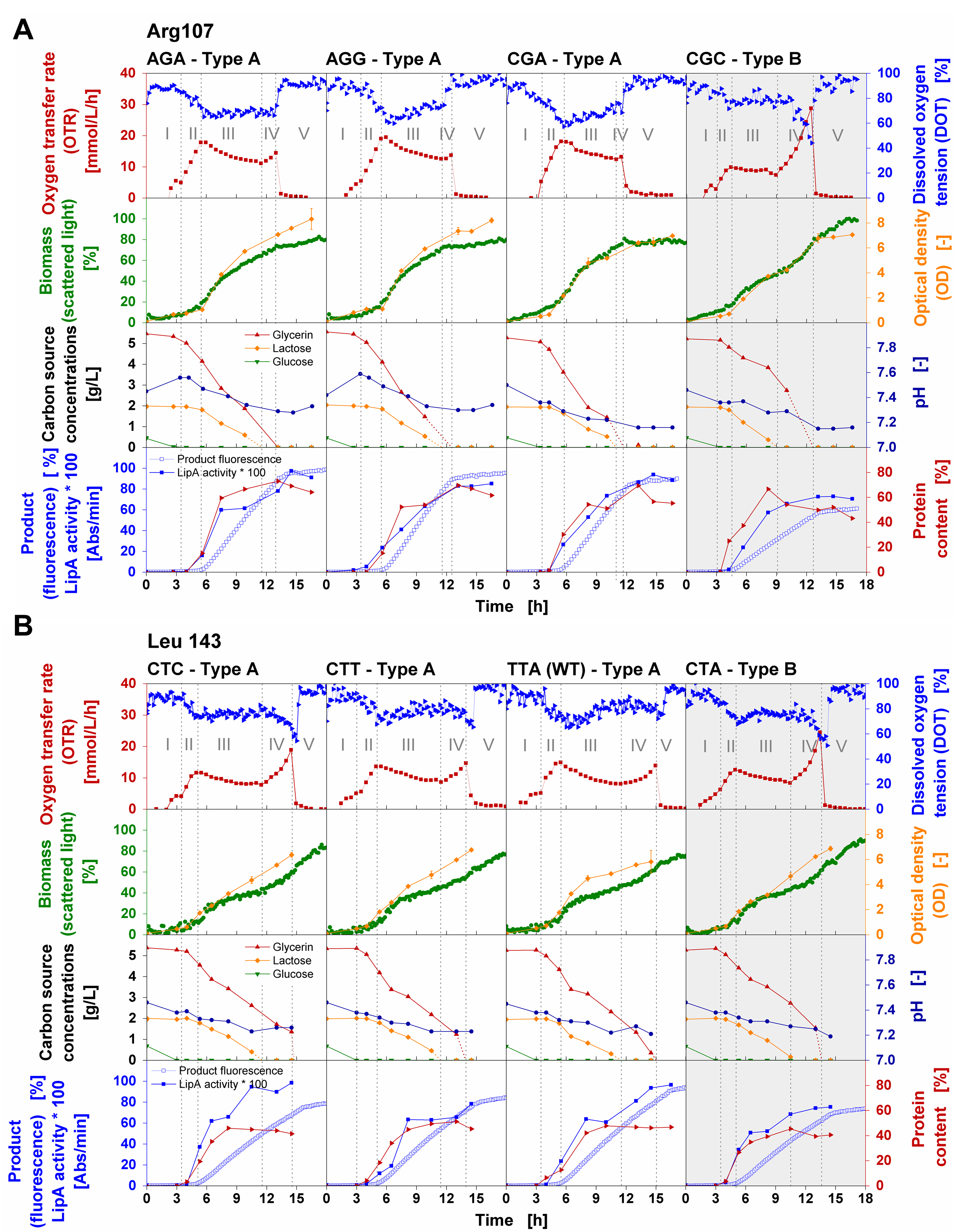

Supplement: Supplementary file 3 — 10.1186/s12934-015-0348-8 Cultivation parameters of eight E. coli BL21(DE3) clones grown under inducing conditions. Characterization of (A) four E. coli BL21(DE3) clones varying in Arg107 codon and (B) four clones varying in Leu143 codon, belonging to respiration behavior Type A (Arg107-AGA, -AGG, -CGA, and Leu143-CTC, -CTT, -TTA (WT); white background) or respiration behavior Type B (Arg107-CGC, Leu143-CTA; grey background) during the cultivation in Wilms-MOPS mineral autoinduction medium containing 0.5 g/L glucose, 5 g/L glycerol and 2 g/L lactose. Cultivation parameters: oxygen transfer rate (OTR, determined using a RAMOS device), biomass (scattered light), soluble product (fluorescence), dissolved oxygen tension (DOT, all determined using a BioLector device), carbon source concentrations, optical density (OD), pH-value, enzyme activity and protein content of the target protein per total protein of the cell (all determined from parallel experiments in conventional shake flasks). The red and orange dotted lines represent the expected depletion of glycerol and lactose, respectively, based on the OTR profile. The vertical grey dotted lines separate the five cultivation phases (I-V) identified by the OTR curves according to Rahmen et al. [23]. Cultivation conditions: 37 °C, 250 mL flasks, filling volume 10 mL, shaking frequency 350 rpm, shaking diameter 50 mm (in RAMOS and conventional flasks); 37 °C, 48-well Flowerplate, filling volume 1 mL, shaking frequency 1000 rpm, shaking diameter 3 mm (in BioLector). [file 12934_2015_348_MOESM3_ESM.tif]

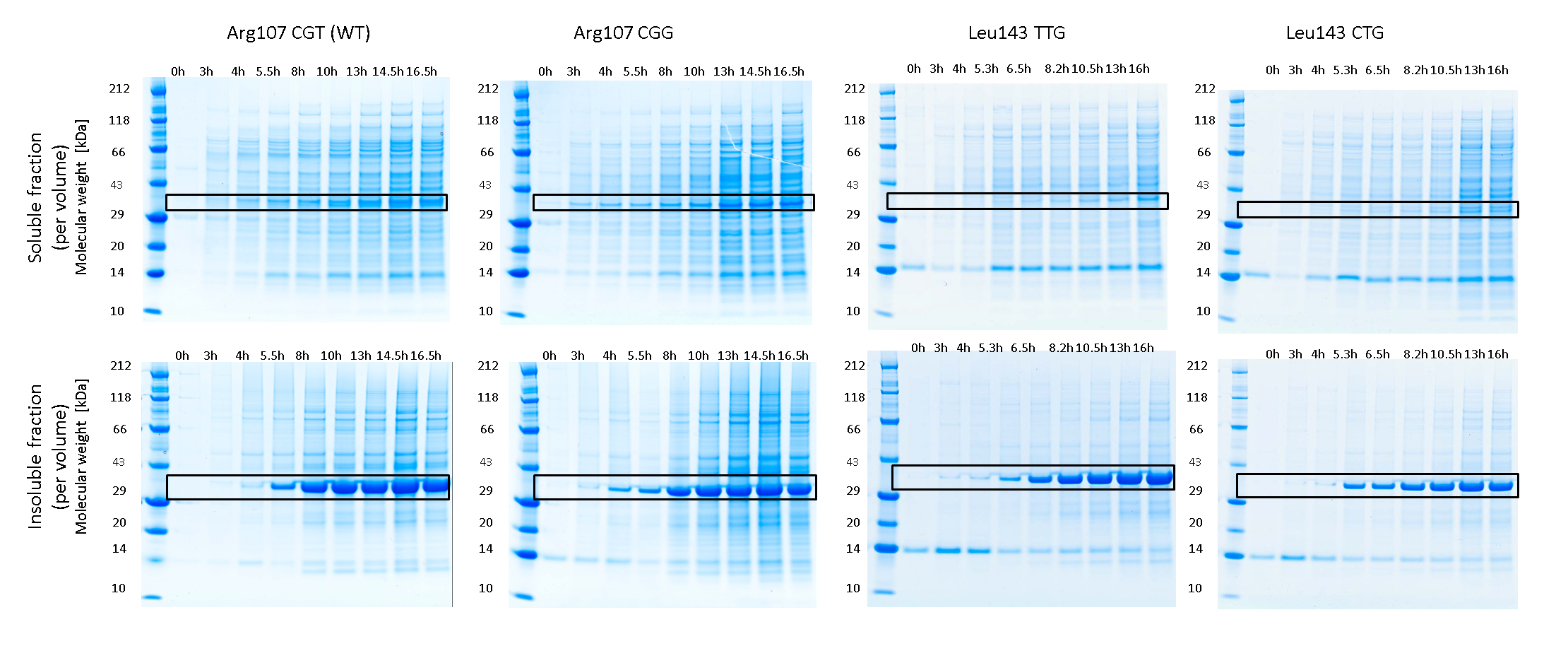

Supplement: Supplementary file 4 — 10.1186/s12934-015-0348-8 SDS-PAGE analysis for soluble and insoluble protein fractions of four clones presented in Figure 4. SDS-PAGE analysis showing soluble (top) insoluble protein (bottom) per sample volume as function of time (target protein band is framed, M = protein marker) for the clones Arg107-CGT (WT, Type A), Arg107-CGG (Type B), Leu143-TTG (Type A) and Leu143-CTG (Type B). [file 12934_2015_348_MOESM4_ESM.jpg]
