# Supplementary material for: A particular silent codon exchange in a recombinant gene greatly influences host cell metabolic activity
Source: Microb Cell Fact. 2015 Oct 5;14:156. doi: 10.1186/s12934-015-0348-8 (PMC4595056; doi:10.1186/s12934-015-0348-8)
Supplement: Supplementary file 6 — 10.1186/s12934-015-0348-8 Supplement to Methods section. [file 12934_2015_348_MOESM6_ESM.docx]

**Additional file 6: Supplement to Methods section**

1. ***Site-directed mutagenesis***

Site-directed silent mutagenesis was applied to introduce the silent mutations into *lipA* gene encoding BSLA. Polymerase chain reactions (PCR) were executed using the modified SPRINP method of Edelheit *et al.* [[52](#_ENREF_52)]. For amplification, two separate 25 µL reactions with each 10-50 ng of template, 0.2 pM of either the forward or reverse primer (synthetized by Eurofins MWG Operon, Germany), 0.2 mM dNTPs, 3 % DMSO (v/v) and 1 U of Phusion high fidelity DNA polymerase in Phusion GC-buffer containing 7.5 mM MgCl_2_ (Thermo Scientific, Germany) were performed. The following PCR program was set: initial denaturation at 98 °C for 10 min, 23 cycles of 98 °C for 1 min, 55 °C for 1 min and 68 °C for 3.5 min, final elongation step at 68 °C for 7 min. PCR was paused after 5 cycles to combine forward and reverse primer reaction and was proceeded for the remaining 18 cycles. Removal of template DNA was achieved using 30 U *Dpn*I at 37 °C for 16 h. After stopping the reaction at 75 °C for 15 min, a PCR purification (Analytik Jena, Germany) was performed. An 1 µL aliquot was transformed into electrocompetent *E. coli* DH5α, plated onto selective Lysogeny Broth (LB) [[62](#_ENREF_62)] agar plates, and incubated overnight at 37 °C. To guarantee successful mutagenesis, positive transformants were sequenced (Eurofins MWG Operon, Germany). Constructed plasmids carrying the silent mutations within BSLA were transformed into competent *E. coli* BL21(DE3) cells and preserved in 15 % (w/w) glycerol at -80 °C.

1. ***Cultivation media***

Complex Terrific Broth (TB) [[53](#_ENREF_53)] medium consists of 12 g/L tryptone, 24 g/L yeast extract, 12.54 g/L K_2_HPO_4_, 2.3 g/L KH_2_PO_4_ and 5 g/L glycerol (all ingredients from Roth, Germany) dissolved in water. The pH-value was 7.2 ± 0.2 without adjustment. Modified Wilms-MOPS mineral medium according to Wilms *et al.* [[54](#_ENREF_54)] consists of 5 g/L glycerol, 0.5 g/L glucose, 5 g/L (NH_4_)_2_SO_4_, 0.5 g/L NH_4_Cl, 3 g/L K_2_HPO_4_, 2 g/L Na_2_SO_4_, 41.85 g/L (N-Morpholino)-propanesulfonic acid (MOPS), 0.5 g/L MgSO_4_·7H_2_O, 0.01 g/L thiamine hydrochloride, 0.1 g/L ampicillin, 1 mL/L trace element solution [0.54 g/L ZnSO_4_·7H_2_O, 0.48 g/L CuSO_4_·5H_2_O, 0.3 g/L MnSO_4_·H_2_O, 0.54 g/L CoCl_2_·6H_2_O, 41.76 g/L FeCl_3_·6H_2_O, 1.98 g/L CaCl_2_·2H_2_O, and 33.4 g/L Na_2_EDTA (Titriplex III)]. The pH-value was adjusted to 7.5 with NaOH. All medium components were sterilized separately by autoclaving or filtration before mixing. Wilms-MOPS mineral autoinduction medium consists of identical ingredients as already mentioned for the Wilms-MOPS mineral medium supplemented with 2 g/L sterilized lactose as inducing compound [[5](#_ENREF_5), [6](#_ENREF_6), [55](#_ENREF_55)].

1. ***Carbon sources***

The concentrations of glucose, lactose, and glycerol were analyzed by HPLC (Ultimate, Dionex, Germany), equipped with an organic acid resin column (250 x 8 mm, CS Chromatographie Service, Germany). 5 mM H_2_SO_4_ was used as eluent at a flow rate of 0.8 mL/min and 60 °C. Resulting peaks were detected via refractive index (Shodex RI-101, Shodwa Denko Europe, Germany).

1. ***Recombinant protein***

Recombinant target protein (BSLA) based on biomass was analyzed via SDS-PAGE. After OD measurement, the culture was centrifuged, the supernatant was removed, the OD was set at 5 by adding a 3:1-mixture of water and four-fold concentrated NuPAGE LDS Sample Buffer (Invitrogen, Germany), and the suspension was shaken at 1000 rpm and 70 °C for 10 min. Afterwards, the SDS-PAGE device (Invitrogen, Germany) was equipped with two gels (4-12 % Bis-Tris, Invitrogen, Germany) and 20 µL of prepared samples and 15 µL of protein marker (Roti-Mark Standard, Roth, Germany) were loaded onto the gel. Running conditions were as follows: running time 35 min, maximum current 200 V, and maximum power 0.25 W. Gels staining was performed overnight under gentle shaking at room temperature in Roti-Blue staining solution (Roth, Germany). Destaining was observed using 25 % methanol for 2 h. Densitometry using the TotalLab TL 100 (TotalLab Ltd, UK) software was performed determining recombinant target protein content relative to total cell protein after electrophoresis.

1. ***Sampling, metabolite extraction and metabolome analysis***

Cells for metabolome analysis were collected according to Izumi *et al.* [[56](#_ENREF_56)]. An appropriate volume of culture broth was transferred to a glass funnel from which culture medium was removed by vacuum suction through a 47 mm diameter, 0.45 µm pore size PTFE membrane filter (Millipore, USA). The volume of culture broth was adjusted based on optical density determined at wavelength 600 nm at respective sampling point to satisfy following formula: sample volume (mL) x OD = 10. Then, filter-bound cells were transferred into a 2 mL microcentrifuge tube (Eppendorf, Germany), and were then rapidly cooled in liquid nitrogen to quench metabolism. The time for the collection process was kept within 30 seconds. Finally, samples were stored at -80°C until extraction.

Metabolite extraction from cells was performed according to Izumi *et al.* [[56](#_ENREF_56)], adding 1.8 mL of extraction solvent (methanol:water:chloroform in 5:2:2 ratio) with camphorsulfonic acid (50 µg/mL) as internal standard. After vortexing the samples, 900 µL of solvent containing extracted metabolites were then transferred into new tubes. A volume of 400 µL of distilled water was added. To separate the polar and nonpolar phases, the mixture was centrifuged at 14000 rpm at 4°C for 5 min. 700 µL of the polar phase hydrophilic metabolites were further transferred to new 1.5 mL microcentrifuge tubes (Eppendorf, Germany). For LC/MS analysis, the polar phase was filtered with a 0.2 µm pore size PTFE filter (Millipore, USA) before transferring into new 1.5 mL tubes. Methanol was removed from the samples by centrifugal concentration for 2 h (SpeedVac, Thermo Scientific, Germany) prior to overnight lyophilization (laboratory freeze dryer VaCo 2, ZIRBUS technology GmbH, Germany). Lyophilized samples were stored at -80°C until analysis.

Cell extracts were analyzed according to Huang *et al.* [[57](#_ENREF_57)] by 1) pentafluorophenylpropyl (PFPP) stationary phase liquid chromatography (Discovery HS F5, 150 mm × 2.1 mm, particle size 3 µm, Sigma-Aldrich Corp., Germany) coupled with electrospray

ionization (ESI) in positive and negative modes, and 2) reversed phase ion-pairing liquid chromatography with a C18 column (CERI L-column2 ODS, 150 mm × 2.1 mm, particle size 3 μm, Chemicals Evaluation and Research Institute, Kyoto, Japan) coupled with ESI in negative mode, to a triple-quadrupole mass spectrometer (LCMS 8030 plus; Shimadzu, Japan). For 1), the mobile phases were 0.1% formic acid (A) and acetonitrile with 0.1 % formic acid (B) at a flow rate of 0.2 mL/min. Concentration of B was increased from 0 % to 40 % and 80 % from 1 to 11 min and 11 to 11.51 min, respectively, held until 12.1 min, decreased to 0% from 12.1 to 12.3 min, and then kept at 0 % until 15 min. Injection volume was 3 μL, and column oven temperature was kept at 40 °C. For 2), the mobile phases were 10 mM tributylamine and 15 mM acetic acid in water (A) and methanol (B) at a flow rate of 0.3 mL/min. Concentration of B was increased from 0 % to 15 % from 1.0 to 2.0 min, kept for 2 min, increased 15 % to 50 %, 55 % and 100 % from 4.0 to 9.0 min, 9.0 to 11.5 min and 11.5 to 12.0 min, respectively, held for 1 min, decreased to 0 % until 13.5 min, and then kept at 0% until 18 min. Injection volume was 3 μL, and column oven temperature was kept at 45 °C. Common MS parameters for both 1) and 2) were as follows: probe position was +1.5 mm, desolvation line temperature was 250 °C, drying gas flow was 15 L/min, and heat block temperature was 400 °C. Nebulizer gas flow was 3 L/min and 2 L/min for 1) and 2), respectively. Other MS parameters were determined by auto-tuning. All analyzed compounds are listed in Additional table 5.1. Compounds highlighted with grey background are presented in Figure 4. Relative intensities of the intracellular compounds were calculated according to the maximum value for each compound after division by the intensity of internal standard.

**Additional table: Analyzed compounds using PFPP chromatography^1^ and ion-pair chromatography^2^**

| **Compound^1^** | **Compound^1^** | **Compound^2^** | **Compound^2^** | **Compound^2^** | **Compound^2^** |
| --- | --- | --- | --- | --- | --- |
| Camphor-sulfonic acid | Thymidine | Arginine | Phenylalanine | F1P | 6-Phospho-gluconate |
| Adenine | Thymine | Histidine | Thymidine | MEP | Oxalacetate |
| Adenosine | Tryptophan | 4-Amino-butyrate | Amino adipic acid | Pyruvate | Isocitrate |
| Alanine | Tyrosine | Serine | Shikimate | DHAP | RuBP |
| Arginine | Uracil | Asparagine | Glycerate | UMP | SBP |
| Asparagine | Uridine | Alanine | Glycolate | GMP | NADH |
| Aspartic acid | Valine | Ornithine | Ascorbate | TMP | Phosphoenol-pyruvate |
| β-Alanine | Xanthine | Inositol | Glyoxylate | AMP | ADP |
| Cysteine |  | Glutamine | G6P | Nicotinate | FMN |
| Cystine |  | Lysine | Disaccharide-P | Pantothenate | HMBPP |
| Cytidine |  | Threonine | Succinicsemi-aldehyde | Succinate | FAD |
| Cytosine |  | Hydroxy-proline | R5P | cAMP | CTP |
| Deoxy-adenosine |  | Cysteine | Pyroglutamate | Glutathione | GTP |
| Deoxycytidine |  | Hexose | Tryptophan | Malate | BPG |
| Deoxy-guanosine |  | 2-Amino-butyrate | S7P | XMP | PRPP |
| Glutamate |  | Disaccharide | Lactate | Acetyl-phosphate | UTP |
| Glycine |  | D-Glucono-1,5-lactone | Guanosine | GDP | NADPH |
| Guanosine |  | Proline | F6P | 2-Oxoglutarate | Camphor-sulfonic acid |
| Histidine |  | Valine | G1P | UDP-Glc | ATP |
| Hypoxanthine |  | Cytidine | α-Glycero-phosphate | CDP | PQQ |
| Isoleucine |  | Methionine | GAP | Fumarate | CoA |
| Leucine |  | Guanine | Thiamine pyrophosphate | 3PGA | Acetoacetyl CoA |
| Lysine |  | Leucine | Orotate | ADP-Glc | 3HB CoA |
| Methionine |  | Tyrosine | E4P | UDP | Succinyl CoA |
| Methionine sulfone |  | Isoleucine | NAD | KDPG | Acetyl CoA |
| Phenylalanine |  | Uridine | Ru5P | NADP | Malonyl CoA |
| Proline |  | Thymine | β-Glycero-phosphate | ADP-Rib | HMG CoA |
| Serine |  | Glutamate | CMP | Citrate | Crotonyl CoA |
| Threonine |  | Inosine | Aspartate | FBP | Butyryl CoA |
